# 2-O-Methylmagnolol, a Magnolol Derivative, Suppresses Hepatocellular Carcinoma Progression via Inhibiting Class I Histone Deacetylase Expression

**DOI:** 10.3389/fonc.2020.01319

**Published:** 2020-08-11

**Authors:** Chi-Yuan Chen, Jia-You Fang, Chin-Chuan Chen, Wen-Yu Chuang, Yann-Lii Leu, Shir-Hwa Ueng, Li-Shan Wei, Shu-Fang Cheng, Chuen Hsueh, Tong-Hong Wang

**Affiliations:** ^1^Tissue Bank, Chang Gung Memorial Hospital, Taoyuan City, Taiwan; ^2^Graduate Institute of Health Industry Technology, Research Center for Food and Cosmetic Safety, Research Center for Chinese Herbal Medicine, College of Human Ecology, Chang Gung University of Science and Technology, Taoyuan City, Taiwan; ^3^Graduate Institute of Natural Products, Chang Gung University, Taoyuan City, Taiwan; ^4^Department of Anesthesiology, Chang Gung Memorial Hospital, Taoyuan City, Taiwan; ^5^Department of Anatomic Pathology, Chang Gung Memorial Hospital, Chang Gung University School of Medicine, Taoyuan City, Taiwan; ^6^Chinese Herbal Medicine Research Team, Healthy Aging Research Center, Chang Gung University, Taoyuan City, Taiwan; ^7^Center for Traditional Chinese Medicine, Chang Gung Memorial Hospital, Taoyuan City, Taiwan; ^8^Department of Hepato-Gastroenterology, Liver Research Center, Chang Gung Memorial Hospital, Taoyuan City, Taiwan

**Keywords:** magnolol, 2-O-methylmagnolol (MM1), histone deacetylase (HDAC), hepatocellular carcinoma (HCC), p21, p53

## Abstract

*Magnolia officinalis* is widely used in Southeast Asian countries for the treatment of fever, headache, diarrhea, and stroke. Magnolol is a phenolic compound extracted from *M. officinalis*, with proven antibacterial, antioxidant, anti-inflammatory, and anticancer activities. In this study, we modified magnolol to synthesize a methoxylated derivative, 2-O-methylmagnolol (MM1), and investigated the use of MM1, and magnolol in the treatment of liver cancer. We found that both magnolol and MM1 exhibited inhibitory effects on the growth, migration, and invasion of hepatocellular carcinoma (HCC) cell lines and halted the cell cycle at the G1 phase. MM1 also demonstrated a substantially better tumor-suppressive effect than magnolol. Further analysis suggested that by inhibiting class I histone deacetylase expression in HCC cell lines, magnolol and MM1 induced p21 expression and p53 activation, thereby causing cell cycle arrest and inhibiting HCC cell growth, migration, and invasion. Subsequently, we verified the significant tumor-suppressive effects of magnolol and MM1 in an animal model. Collectively, these findings demonstrate the anti-HCC activities of magnolol and MM1 and their potential for clinical use.

## Introduction

The World Health Organization (WHO) indicated that liver cancer was the sixth most common cancer and the fourth leading cause of cancer deaths worldwide in 2018, with a global death toll of 782,000 (1). The risk factors for liver cancer include hepatitis B, hepatitis C, alcoholic liver disease, non-alcoholic fatty liver disease, and cirrhosis (2, 3). At present, surgery remains the first line of treatment for liver cancer; however, chemotherapy or radiation therapy is the preferred choice for patients with advanced liver cancer who cannot undergo surgical resection (4). Most chemotherapeutic drugs, however, often have large side effects and significantly impact patients' quality of life (5). Therefore, the development of effective therapeutic drugs with minimal side effects has been at the forefront of liver cancer research.

Due to its advantages, such as high specificity and low side effects, targeted therapy has become the main modality of cancer treatment (6, 7). However, carcinogenic factors are multifactorial and often complicated. This complexity is further aggravated by tumor heterogeneity (8, 9). Therefore, drugs against a single target often demonstrate limited efficacy. Even sorafenib, which is recognized as the most effective targeted drug against liver cancer, only prolongs patient survival by ~3 months (10, 11). Thus, in clinical practice, targeted therapy is often used in conjunction with other treatment modalities, such as chemotherapy and radiation therapy, to improve therapeutic outcomes (12, 13).

Recent studies have shown that the occurrence of liver cancer is closely associated with genetic and epigenetic variations (14, 15). Common epigenetic regulatory mechanisms include DNA methylation, histone modification, and non-coding RNA regulation (16). Previous studies have reported that histone deacetylase (HDAC) overexpression is common in hepatitis B virus (HBV)-infected liver cancer patients (17, 18) and could lead to carcinogenesis, as HDACs regulate the deacetylation of histone and non-histone proteins, thereby coordinating gene expression or protein activation. Histone protein deacetylation leads to its tighter binding of the surrounding DNA, consequently inhibiting gene expression in the bound region. Alternatively, non-histone protein acetylation not only is closely associated with its protein activity but also affects its ability to bind other proteins or DNA, thereby indirectly regulating the expression of other genes and their encoded proteins (19, 20). The 18 known HDAC types found in humans can be categorized into four classes: class I (HDAC1, HDAC2, HDAC3, and HDAC8), class IIa (HDAC4, HDAC5, HDAC7, and HDAC9), class IIb (HDAC6 and HDAC10), class III Sir2-like enzymes (comprising seven sirtuins), and class IV (HDAC11). Among these, class I HDAC overexpression is observed in most cancer types, including liver cancer (21–24). Class I HDAC overexpression can inhibit the expression of multiple tumor-suppressor genes, such as p21 and p53, thereby promoting carcinogenesis (25–27). Moreover, these HDACs are therapeutic targets for multiple anticancer treatments. HDAC inhibitors, including trichostatin A, vorinostat (suberoylanilide hydroxamic acid, SAHA), trapoxin A, and valproic acid, are effective in the treatment of lung, breast, and esophageal cancers, whereas, SAHA has been approved by the Food and Drug Administration (FDA) for the treatment of T-cell lymphoma (28–31). Furthermore, recent studies have found that the combined use of an HDAC inhibitor with sorafenib can substantially improve the treatment efficacy of sorafenib in liver cancer (32, 33). However, most HDAC inhibitors have significant side effects, which are the reason for the primary bottleneck to their clinical use.

The application of traditional Chinese herbal medicine in disease treatment has become increasingly popular in recent years. Compared to Western medicine, Chinese herbal medicine is an alternative treatment option that can be effective and introduces fewer side effects (34–36). Owing to the development of component separation technologies, the active ingredients of traditional Chinese medicines have been extracted and their functions identified. These compounds can act at lower effective doses and produce more specific therapeutic effects. Among them, artemisinin and curcumin are used and have shown good outcomes in cancer treatment (37–39). Other extracts, such as resveratrol and chrysin, exert an anti-cancer stem cell (CSC) effect and may provide an alternative approach to manage cancers (40).

*Magnolia officinalis* is a traditional Chinese medicinal plant commonly used in Southeast Asian countries. Its extract, magnolol, a phenolic compound, has proven antibacterial, antioxidant, and anti-inflammatory activities, and its anticancer and antiangiogenic activities have also been recently verified (41, 42). However, the mechanism of its anticancer effects is yet to be elucidated. In the present study, we modified magnolol and synthesized a methoxylated derivative, 2-O-methylmagnolol (MM1). In addition to testing the anti-hepatocellular carcinoma (HCC) activities of magnolol and its derivative MM1, we also used cell and animal models to clarify their modes of action, thereby elucidating the feasibility of their clinical applications.

## Materials and Methods

### Cell Culture

Human HCC cell lines Huh7 and HepG2 were purchased from the American Type Culture Collection (Manassas, VA, USA) and donated by Dr. Chau-Ting Yeh of Chang Gung Memorial Hospital, respectively. Human skin fibroblasts (HFBs) were kindly provided by Dr. Pan-Chyr Yang of Taiwan University. The cells were maintained in Dulbecco's Modified Eagle Medium (Gibco, Gaithersburg, MD, USA) containing 10% fetal bovine serum (FBS) and cultured at 37°C with 5% carbon dioxide in a humidified incubator. Culture medium, chemical compounds, and FBS were purchased from Life Technologies (Grand Island, NY, USA).

### Compounds and Antibodies

Magnolol was purchased from Shanghai BS Bio-Tech Co., Ltd. (Shanghai, China). MM1 was prepared as described by Lin et al. (43). The purity of magnolol and MM1 was <99%, as determined by high-precision liquid chromatography (HPLC) analysis. Magnolol and MM1 were each dissolved in dimethyl sulfoxide (DMSO) to obtain a stock concentration of 100 mM, which was then stored at −20°C before use. DMSO 0.1% v/v was used as the vehicle control. Sorafenib was purchased from Sigma-Aldrich (St. Louis, MO, USA). Antibodies against human class I HDACs (HDAC1, HDAC2, HDAC3, and HDAC8), acetyl-histone H3, acetyl-p53, p53, p21, Ki-67, E-cadherin, N-cadherin, vimentin, Snail, Slug, and β-actin were purchased from GeneTex (Irvine, CA, USA) and Cell Signaling Technology (Beverly, MA, USA). The antibody to cyclin D1 was purchased from ABclonal Technology (Woburn, MA, USA), and the antibodies against CDK4 were purchased from Proteintech (Rosemont, IL, USA). Secondary antibodies were purchased from Santa Cruz Biotechnology (Santa Cruz, CA, USA).

### Real-Time Reverse Transcription-Polymerase Chain Reaction Analysis

Total RNA from Huh7 and HepG2 cells were extracted using TOOLSmart RNA extractor (BIOTOOLS Co., Ltd., Taiwan) and RNeasy Mini Kit (QIAGEN, Gaithersburg, MD, USA) according to the manufacturer's instructions. Complementary DNA was synthesized using a ToolScript MMLV RT Kit (BIOTOOLS Co., Ltd., Taiwan). Quantitative real-time polymerase chain reaction (PCR) assays using the TaqMan Gene Expression Kit (Applied Biosystems, Foster City, CA, USA), TOOLS 2 × SYBR qPCR Mix (BIOTOOLS Co., Ltd., Taiwan), and an ABI StepOnePlus™ System (Applied Biosystems) were used to detect p21 expression, using glyceraldehyde 3-phosphate dehydrogenase as an internal control.

### Western Blot Analysis

Huh7 and HepG2 cells were treated with magnolol, MM1, or dimethyl sulfoxide (DMSO) for 48 h, followed by lysis in RIPA lysis buffer (BIOTOOLS Co., Ltd., Taiwan) containing protease inhibitors. Cell lysates (30-μg protein) were subjected to Western blotting as described previously, using β-actin as a loading control. The relative intensities of the protein bands were quantified using ImageQuant 5.2 software (GE Healthcare, Piscataway, NJ, USA).

### *In vitro* Cell Proliferation Assay

The proliferation capacity of magnolol-/MM1-treated cells was examined using an xCELLigence Real-Time Cell Analyzer (Roche Life Science, Indianapolis, IN, USA) according to the manufacturer's standard protocol.

### Transwell Migration and Invasion Assay

The migration and invasion capacities of magnolol-/MM1-treated cells were analyzed using a Transwell migration assay, as described previously (44).

### Cell Cycle Analysis

Cells were trypsinized, washed twice, and fixed with 70% ethanol at −20°C for 1 h. The fixed cells were subsequently incubated in phosphate-buffered saline containing 0.12% Triton X-100, 0.12 mmol/L ethylenediaminetetraacetic acid, and 100 mg/mL ribonuclease A at 37°C for 1 h. Cells were stained with propidium iodide (50 μg/mL) at 4°C for 20 min, and cell cycle distribution was measured using a BD FACS caliber.

### Cell Apoptosis Assay

The apoptosis status of Huh7 cells was determined using a DeadEnd™ Fluorometric terminal deoxynucleotidyl transferase dUTP nick end labeling (TUNEL) assay kit (Promega, Madison, WI, USA) according to the manufacturer's protocol. In summary, Huh7 cells were grown on chamber slides and treated with different concentrations of magnolol or MM1 for 48 h. The cells were fixed with 4% paraformaldehyde for 15 min at room temperature and subsequently subjected to the TUNEL assay. Apoptotic cells were examined using a fluorescence microscope (magnification × 100). Images of five random fields per dish were examined for each experiment.

### Tumor Formation Assay in Nude Mice

Six-week-old male BALB/c nude mice were purchased from the National Laboratory Animal Center (Taipei, Taiwan), and maintained under specific pathogen-free conditions. Animal experiments were performed under an approved protocol in accordance with the guidelines for the Animal Care and Ethics Commission of Chang Gung Memorial Hospital (IACUC Approval No. 2018031301; approval date: 6/19/2018). The mice were injected subcutaneously with 5 × 10^6^ Huh7 cells (in 100 μL of saline with 50% Matrigel [BD Biosciences]) into both flanks. All tumors were allowed to grow for 1 week before the initiation of drug treatment. At the start of the second week, mice with tumors were intraperitoneally injected three times a week with 100 μL of magnolol or MM1 (0.1 μmol in 100 μL of DMSO) or an equal volume of DMSO, which served as a control. Twenty-eight days after drug administration, the mice were euthanized and the tumors were subjected to immunohistochemical staining.

### Immunohistochemistry

The tumors from the mice were fixed in 4% paraformaldehyde overnight, dehydrated, and embedded in paraffin. Paraffin blocks were sliced into 2-mm-thick sections and floated onto glass slides. The tissue sections were deparaffinized, and the expression of HDAC1, HDAC2, p21, cyclin D1, CDK4, Ki-67, E-cadherin, N-cadherin, vimentin, and Snail in the tissues were detected as described previously (45).

### Statistical Analyses

Comparisons between groups were analyzed using Student's *t*-tests. The results are expressed as the mean ± standard deviation. The half-maximal inhibitory concentration (IC_50_) values were determined by non-linear regression analysis using GraphPad Prism version 8.0 (GraphPad Software Inc., La Jolla, CA, USA). All statistical analyses were performed using the Statistical Package for the Social Sciences version 16.0 and Microsoft Excel 2007. All *p*-values were two-sided, with *p* < 0.05 considered to indicate a statistically significant difference.

## Results

### 2-O-Methylmagnolol (MM1) Has Superior Inhibitory Effects on Hepatocellular Carcinoma (Hcc) Cell Growth, Metastasis, and Invasion

**Figure 1:**
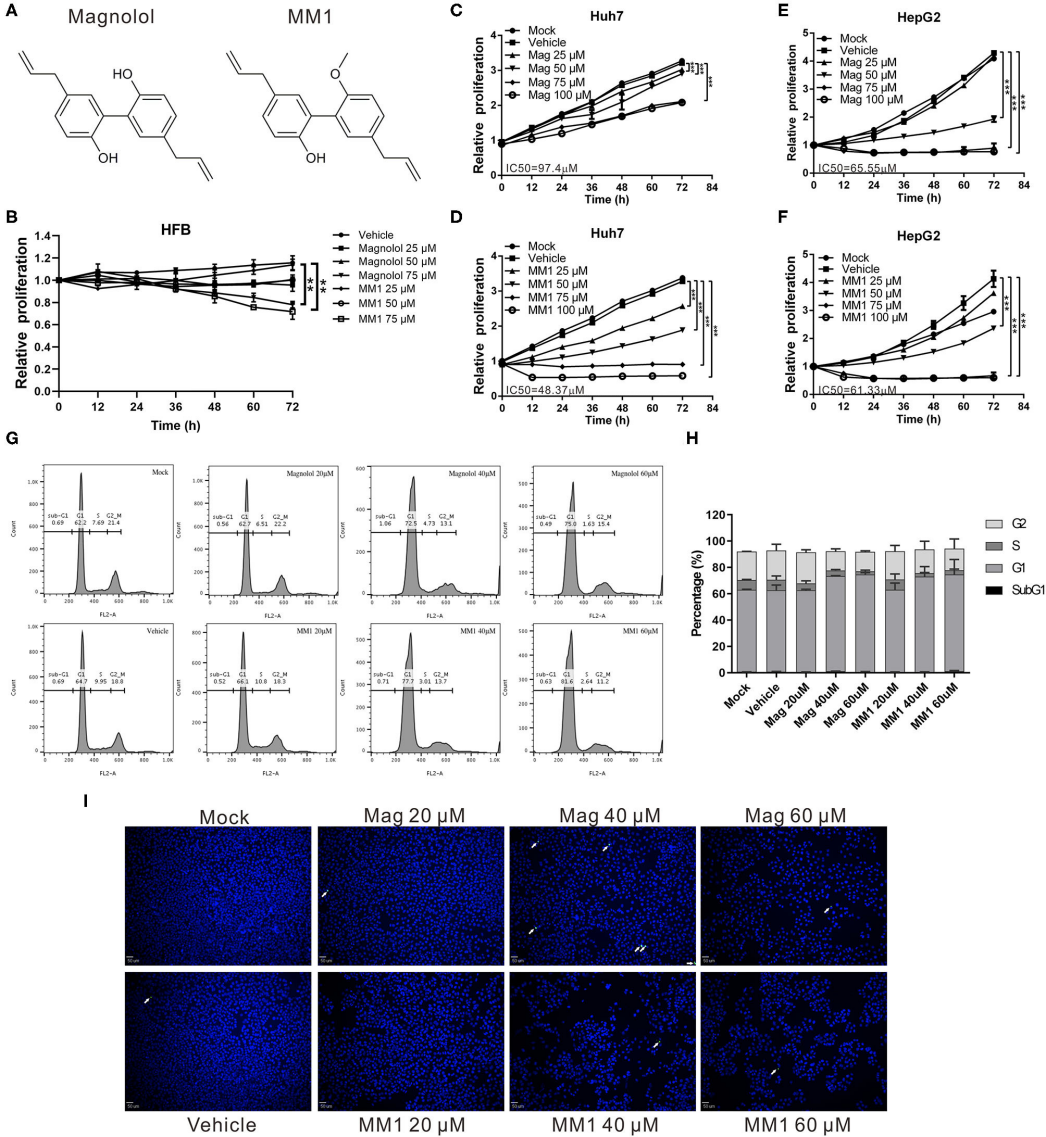
Compared to magnolol, 2-O-methylmagnolol (MM1) demonstrates a greater ability to inhibit hepatocellular carcinoma (HCC) cell growth. **(A)** Chemical structures of MM1 and magnolol. **(B–F)** Human skin fibroblasts, Huh 7, and HepG2 cells were treated with different concentrations (0, 25, 50, 75, and 100 μM) of magnolol or MM1, and the cell proliferation status was analyzed using an xCELLigence Real-Time Cell Analyzer. The results are shown as the mean ± standard deviation of three independent experiments. Significant differences compared with the vehicle groups, **p < 0.01, ***p < 0.001. **(G)** Effect of magnolol and MM1 on cell cycle progression in Huh7 cells. Cells were treated with the indicated concentrations of magnolol or MM1 for 48 h. Cell cycle distribution was measured by propidium iodide staining and quantified by flow cytometry. The quantitative results are shown in **(H)**. **(I)** Effects of magnolol and MM1 on apoptosis in Huh7 cells. Terminal deoxynucleotidyl transferase dUTP nick end labeling (TUNEL) staining was used to observe the apoptotic cells under a fluorescence microscope (magnification × 100). Green punctate staining represents TUNEL-positive cells (white arrow).

To determine whether magnolol and MM1 exhibited anticancer activities against liver cancer (Figure 1A), HCC cell lines, HepG2, and Huh7, were treated with different concentrations of magnolol and MM1 to analyze their effects on cell growth. The results suggested that both magnolol and MM1 significantly inhibited HCC cell growth. Compared to the control group treated with DMSO, magnolol inhibited the growth of the two cell lines from 25 μM onward, with increasing effects in a dose-dependent manner. However, MM1 displayed a significantly stronger inhibitory effect on cell growth than magnolol at similar concentrations, indicating a greater tumor-suppressive activity than that of magnolol (Figures 1C–F). The half-maximal inhibitory concentration (IC_50_) of magnolol toward Huh7 and HepG2 cells was ~97 and 65 μM, respectively, which is similar to results from other studies (46–48), while the IC_50_ of MM1 in Huh7 and HepG2 cells was 48 and 61 μM, respectively. Moreover, only a slight inhibitory effect was observed on the growth of the HFB cell line at the highest concentrations of magnolol and MM1 (Figure 1B). This finding indicated that magnolol and MM1 selectively inhibited HCC cell growth with low toxicity to normal cells.

Flow cytometry analysis to further understand the potential influences of magnolol and MM1 on the cell cycle showed that treatment with magnolol and MM1 caused cells to stagnate at the G1 phase (Figures 1G,H). Additionally, even at high concentrations, magnolol, and MM1 treatment did not cause apoptosis (Figure 1I). These findings suggest that magnolol and MM1 inhibited cell growth by causing cell cycle arrest.

**Figure 2:**
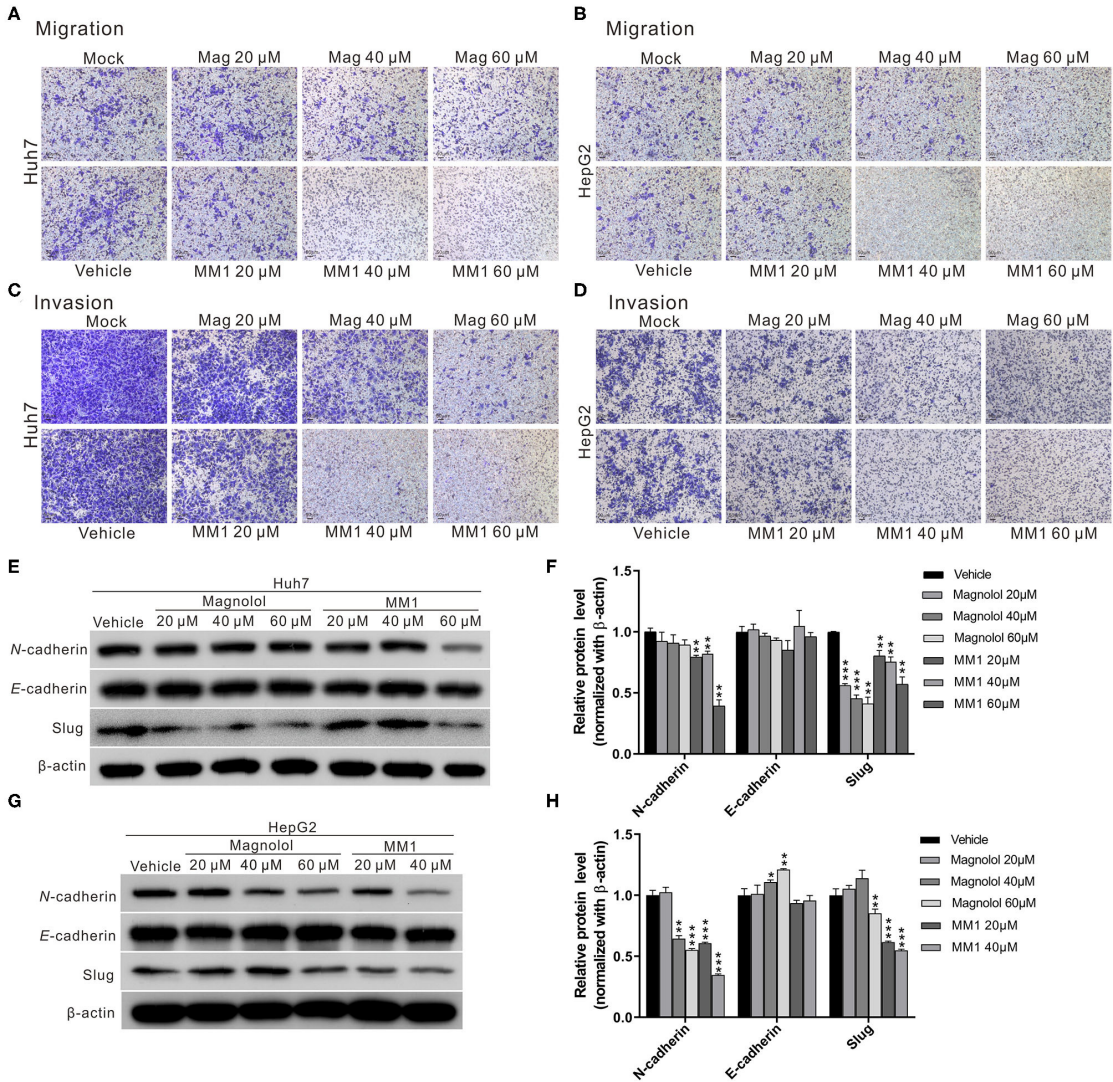
2-O-methylmagnolol (MM1) and magnolol inhibit hepatocellular carcinoma cell migration and invasion by suppressing epithelial-mesenchymal transition (EMT). **(A,B)** Comparisons of migration capacities of Huh7 and HepG2 cells treated with magnolol or MM1 in transwell assays. **(C,D)** Invasion assays using Matrigel-coated polyethylene terephthalate membrane inserts. **(E,G)** Western blotting showing the expression of EMT-related proteins in Huh7 and HepG2 cells after treatment with magnolol and MM1. Quantitative results are shown in **(F,H)**. The results are shown as the mean of three independent experiments. Significant differences compared with the vehicle control groups, *p < 0.05, **p < 0.01, ***p < 0.001.

One of the primary reasons that liver cancer is difficult to cure is the strong invasion and metastasis ability of tumor cells. To investigate the effects of magnolol and MM1 on the metastasis and invasion ability of HCC cells, we performed a transwell migration assay. The results indicated that both magnolol and MM1 had inhibitory effects on cell migration ability (Figures 2A,B). Similar inhibitory effects were also observed on the invasion abilities of HCC cells at similar concentrations (Figures 2C,D). Consistent with the results of the cell growth analysis, MM1 displayed higher inhibitory effects on the migration and invasion capacities of HCC cells, compared to those of magnolol at similar concentrations.

Considering that the epithelial-mesenchymal transition (EMT) is an important process for tumor metastasis, we also measured the expression levels of EMT-related proteins such as N-cadherin, E-cadherin, and slug to determine whether magnolol and MM1 inhibited HCC migration and invasion by regulating EMT. The results showed significantly lower expression of EMT-promoting proteins, N-cadherin, and slug in magnolol and MM1-treated cells compared to that in the control group (Figures 2E–H). These findings suggested that magnolol and MM1 inhibited HCC migration and invasion by suppressing EMT.

### Magnolol and MM1 Inhibit Class I Histone Deacetylase Expression in HCC Cells

**Figure 3:**
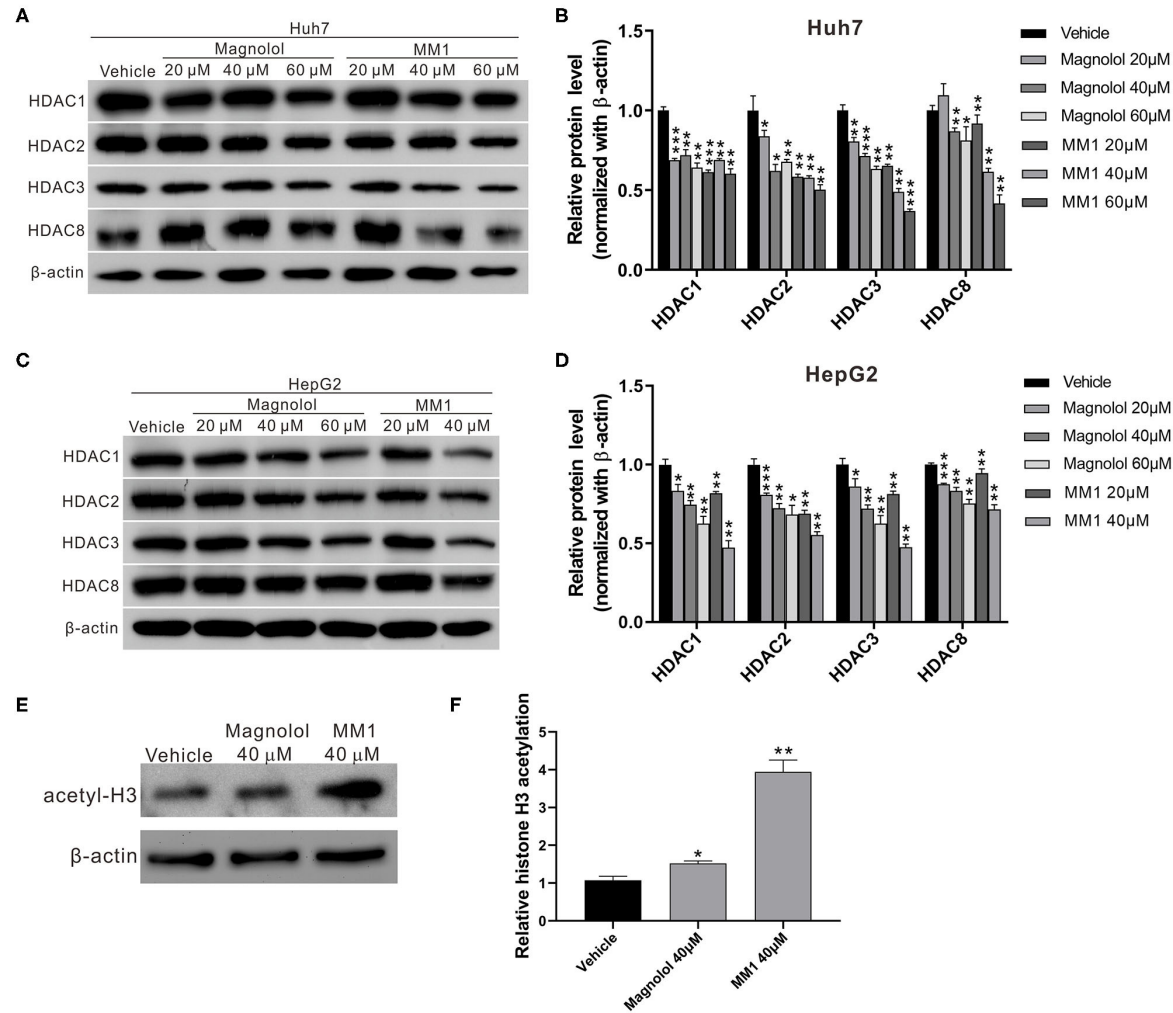
2-O-methylmagnolol (MM1) and magnolol inhibit class I histone deacetylase (HDAC) expression in hepatocellular carcinoma cell lines. **(A,C)** Huh7 and HepG2 cells were treated with magnolol, MM1, or vehicle for 48 h. The expression levels of HDACs 1, 2, 3, and 8 were determined by Western blotting. Quantitative results are shown **(B,D)**. **(E)** The levels of acetylated histone H3 in HepG2 cells were examined by Western blotting. The quantitative results are shown in **(F)**. The measurement data are expressed as the mean ± standard deviation of three independent experiments. *p < 0.05, **p < 0.01, ***p < 0.001.

Previous studies suggest that magnolol could inhibit non-small cell lung cancer progression by inhibiting class I HDAC expression (49). Additionally, the overexpression of class I HDACs commonly observed in liver cancer patients is associated with liver cancer progression (32). To determine whether the anti-HCC effects of magnolol and MM1 were exerted by inhibiting class I HDACs, Western blot analysis was performed to examine the expression of class I HDACs in HCC cells treated with magnolol and MM1. The results indicated that treatment with magnolol and MM1 considerably inhibited the expression of HDAC 1, 2, 3, and 8 proteins. Additionally, the inhibitory effect of MM1 on class I HDACs was significantly higher than that of magnolol at similar concentrations (Figures 3A–D). Another Western blot analysis performed to investigate the association between magnolol or MM1 treatment and the degree of acetylation of histone H3 in HCC cell lines showed substantially higher histone H3 acetylation in cells treated with magnolol and MM1 compared to that in the control group (Figures 3E,F). These findings indicated that magnolol and MM1 promoted histone acetylation by inhibiting HDAC expression.

### Magnolol and MM1 Induce p21 Expression and p53 Acetylation

**Figure 4:**
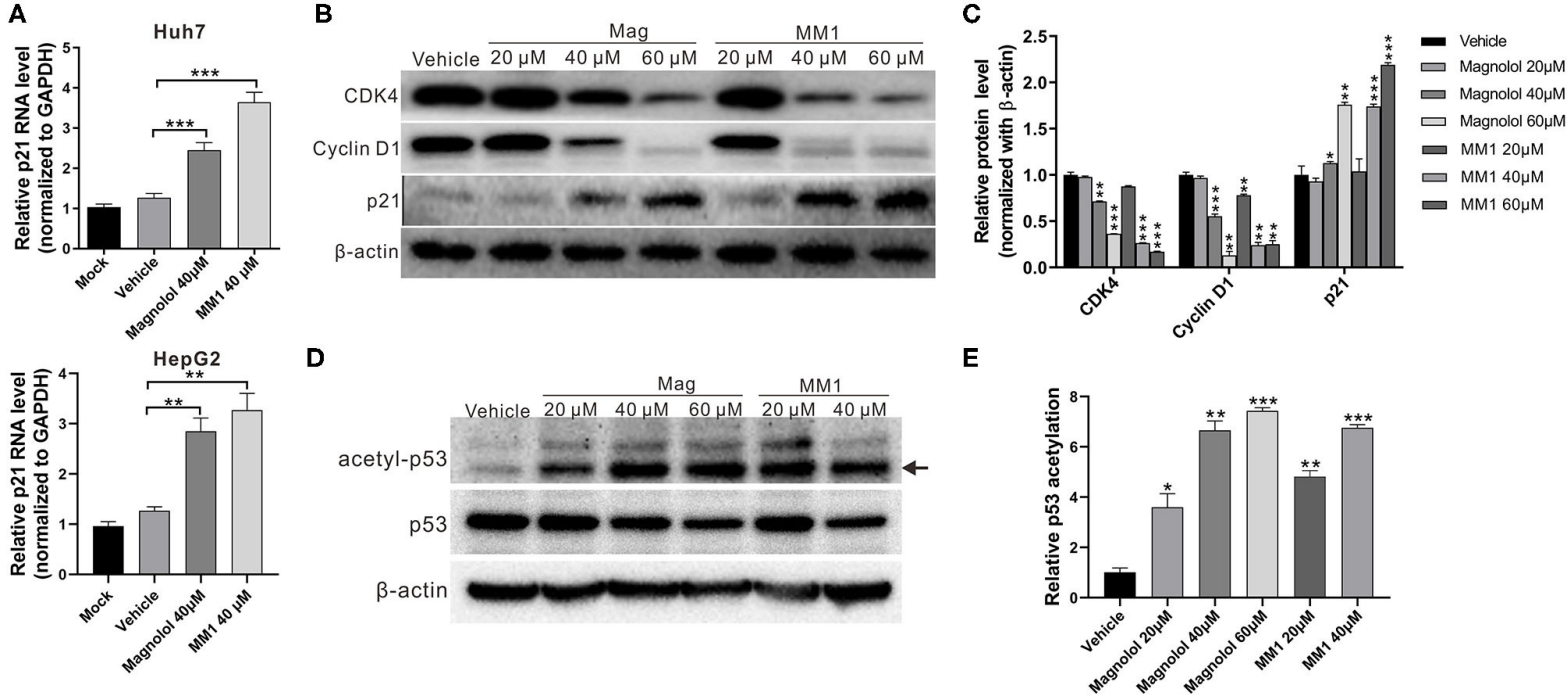
2-O-methylmagnolol (MM1) and magnolol induce the expression of the tumor-suppressor gene p21 and the acetylation of p53. **(A)** Huh7 and HepG2 cells were treated with the indicated concentrations of magnolol or MM1 for 48 h. The p21 RNA levels were examined by quantitative real-time reverse transcription-polymerase chain reaction. **(B)** Expression levels of p21 and downstream proteins in Huh7 cells were analyzed by Western blot using β-actin as an internal control. Quantitative results are shown in **(C)**. *p < 0.05, **p < 0.01, ***p < 0.001. **(D)** HepG2 cells were treated with magnolol or MM1 for 48 h, and the levels of acetylated p53 were examined by Western blot. Quantitative results are shown in **(E)**. Error bars represent mean ± standard deviation from three independent experiments. *p < 0.05, **p < 0.01, ***p < 0.001.

HDACs can regulate the degree of deacetylation of histone and non-histone proteins, thereby suppressing gene expression. Previous studies have reported that class I HDACs induce carcinogenesis by inhibiting the expression of the tumor-suppressor gene p21 and activating the tumor-suppressor protein p53 (26, 27). Thus, real-time RT-PCR and Western blot analyses were performed to identify the effects of magnolol and MM1 on the expression and activation of p21 and p53. The results showed substantially higher expression of p21 mRNA and protein in Huh7 and HepG2 cells treated with magnolol and MM1 and lower expression of cell cycle regulatory proteins such as CDK4 and cyclin D1 than in the control group (Figures 4A–C). These findings suggested that magnolol and MM1 could induce p21 gene expression, thereby impeding cell cycle progression. Furthermore, the fact that the degree of p53 protein acetylation increased with increasing magnolol and MM1 concentrations (Figures 4D–E) suggested that magnolol and MM1 could promote the activation of p53 tumor-suppressor proteins.

### Magnolol and MM1 Enhance the Anti-HCC Effect of Sorafenib

**Figure 5:**
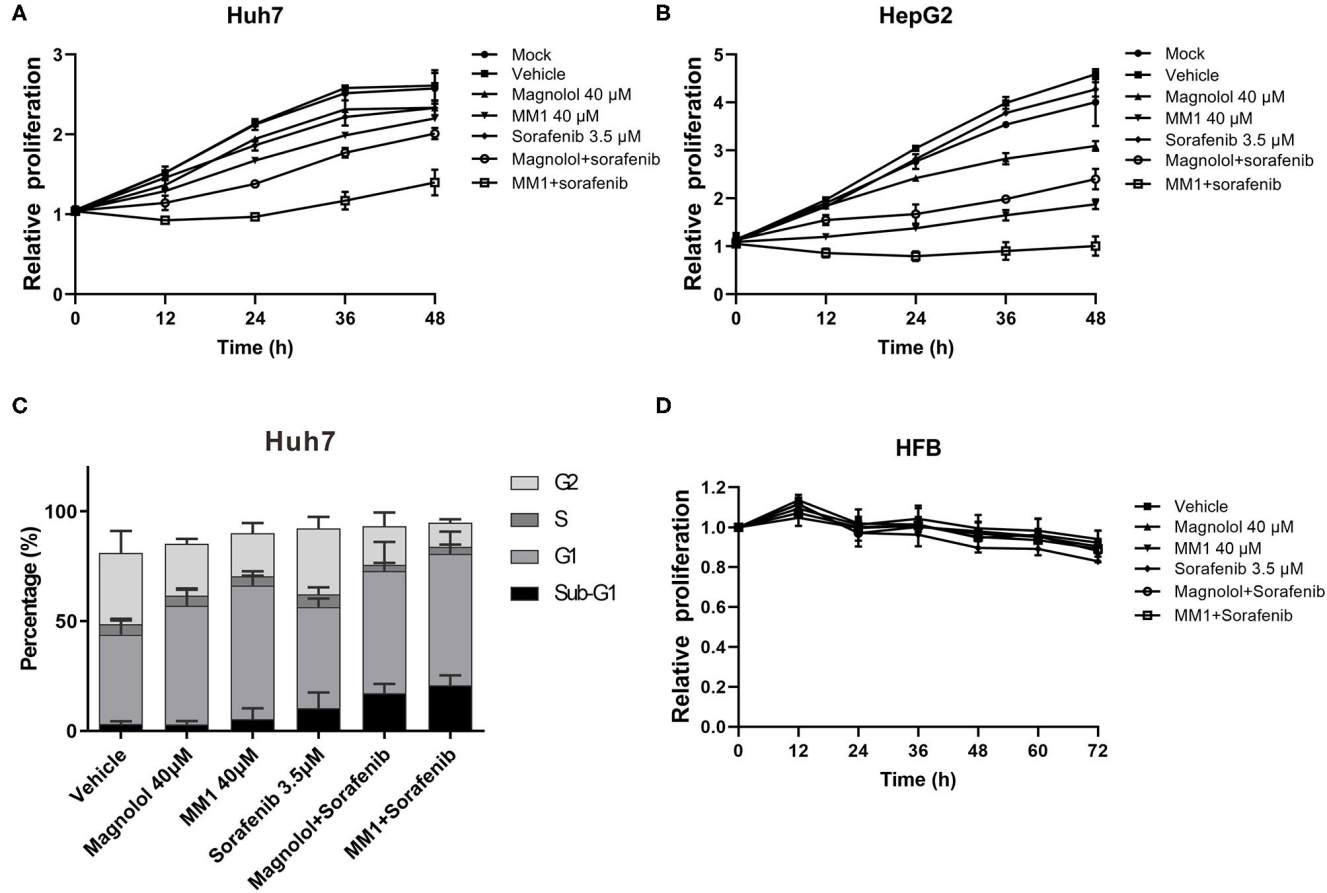
2-O-methylmagnolol (MM1)/magnolol and sorafenib show a synergistic anti-hepatocellular carcinoma effect. **(A,B)** Huh7 and HepG2 cells were treated with 40 μM magnolol/MM1 and 3.5 μM sorafenib, individually, or in combination. The cell proliferation status was analyzed using an xCELLigence Real-Time Cell Analyzer. The data are expressed as the mean ± standard deviation from three independent experiments. **(C)** The cell cycle status in Huh7 cells treated with magnolol/MM1/sorafenib was examined by flow cytometry. **(D)** Effects of magnolol/MM1 and sorafenib alone or in combination on cell proliferation in human fibroblasts.

Previous studies suggest that the combined use of HDAC inhibitors and sorafenib could enhance the antitumor effect of sorafenib (32, 33). To understand whether the combined use of magnolol/MM1 and sorafenib showed compounded effects, magnolol/MM1 and sorafenib were administered individually and concurrently to HCC cell lines. The cell proliferation assay and flow cytometry were performed to analyze cell proliferation and cell cycle progression. The results indicated that individual treatment with magnolol/MM1 or sorafenib led to cell stagnation at the G1 phase and induced cell apoptosis. In contrast, the concurrent administration of magnolol/MM1 and sorafenib substantially improved the toxic effect on HCC cell lines (Figures 5A–C). These findings verified that the combined use of magnolol and sorafenib could enhance the efficacy of anti-liver cancer treatment.

To understand the safety of the combined use of magnolol/MM1 and sorafenib, the effects of the above compounds on human fibroblasts HFB alone or in combination were tested (Figure 5D). We found that magnolol and MM1 did not significantly affect the growth of HFB, whereas sorafenib slightly inhibited the growth of HFB. However, when MM1 or magnolol are used in combination with sorafenib, it can reduce the toxicity of sorafenib to HFB.

### Magnolol and MM1 Inhibit Tumor Growth in Mice

**Figure 6:**
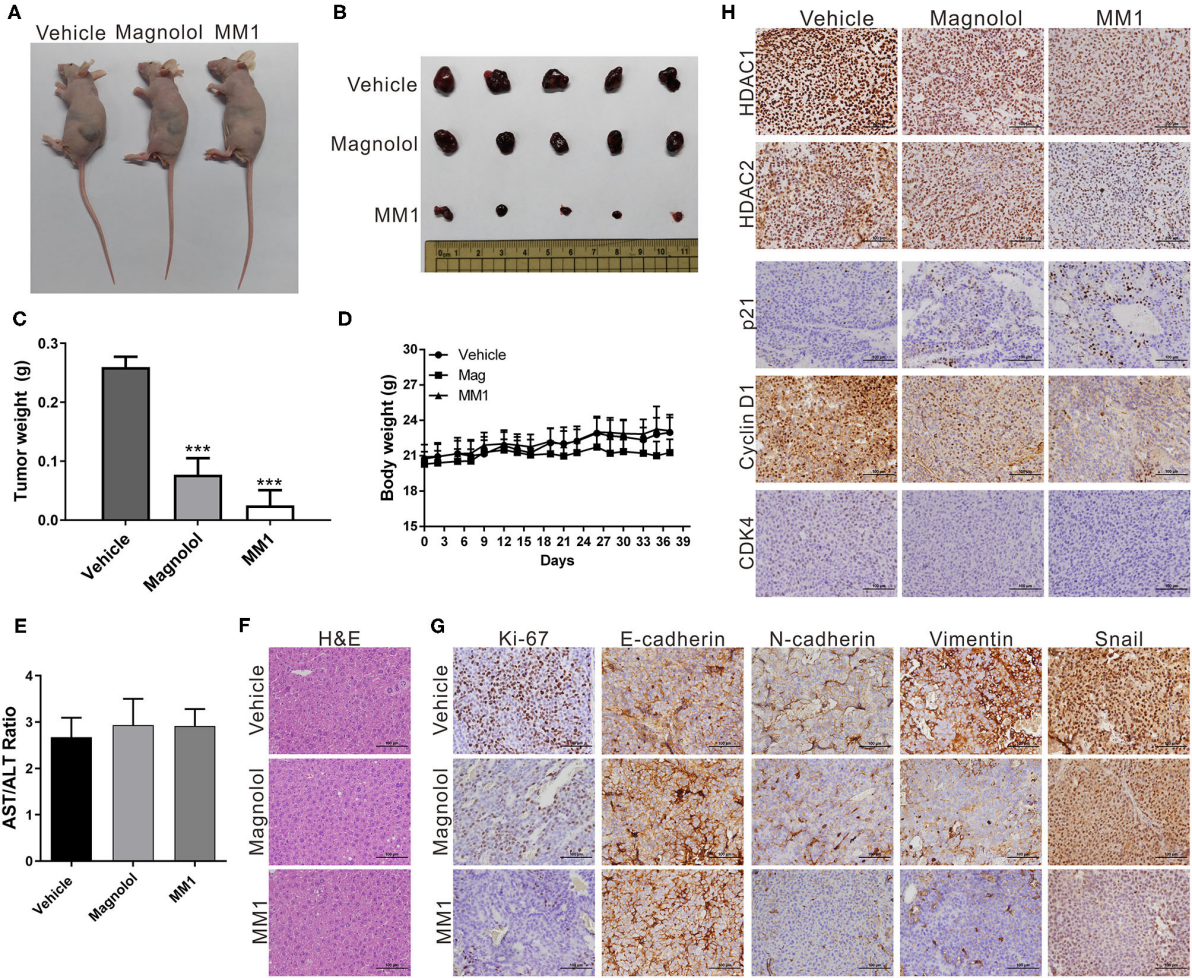
2-O-methylmagnolol (MM1) and magnolol inhibit tumor growth in mice. **(A)** A total of 5 × 10^6^ Huh7 cells were injected into the dorsal flanks of nude mice (*n* = 5 per group). Subsequently, the mice were intraperitoneally injected three times per week with 100 μL of magnolol or MM1 [0.1 μmol in 100 μL of dimethyl sulfoxide (DMSO)] or an equal volume of DMSO. Representative images show the tumor xenografts at 5 weeks post-implantation. **(B)** Tumor tissues were collected at the end point. **(C)** Tumor weights at end point. **(D)** Body weights measured during the experiment. ***p < 0.001. **(E)** Serological test results of the three groups of mice. **(F)** Hematoxylin and eosin (H&E) staining of mouse liver tissue sections. Magnification: 400×. **(G,H)** Immunohistochemical staining showing the effect of magnolol or MM1 on class I histone deacetylases, p21, CDK4, cyclin D1, Ki-67, and EMT-related protein expression in mouse xenograft tumors. Magnification: 400×.

To confirm that magnolol and MM1 demonstrated the same inhibitory effects on HCC cells *in vivo* and verified the abovementioned regulatory mechanism, a mouse xenograft model was established by injecting Huh7 cells into the backs of mice and subsequently administering magnolol or MM1 periodically via intraperitoneal injection. The results suggested that, compared to the control group that only received DMSO, the administration of either magnolol or MM1 significantly inhibited tumor growth in mice. In addition, the inhibitory effect of MM1 was superior to that of magnolol (Figures 6A–C). Furthermore, the weights and liver tissue morphology of mice treated with magnolol or MM1 did not change considerably, nor were there any significant abnormalities in the serological test results for the two groups of mice (Figures 6D–F), indicating that neither treatment was toxic to the mice.

**Figure 7:**
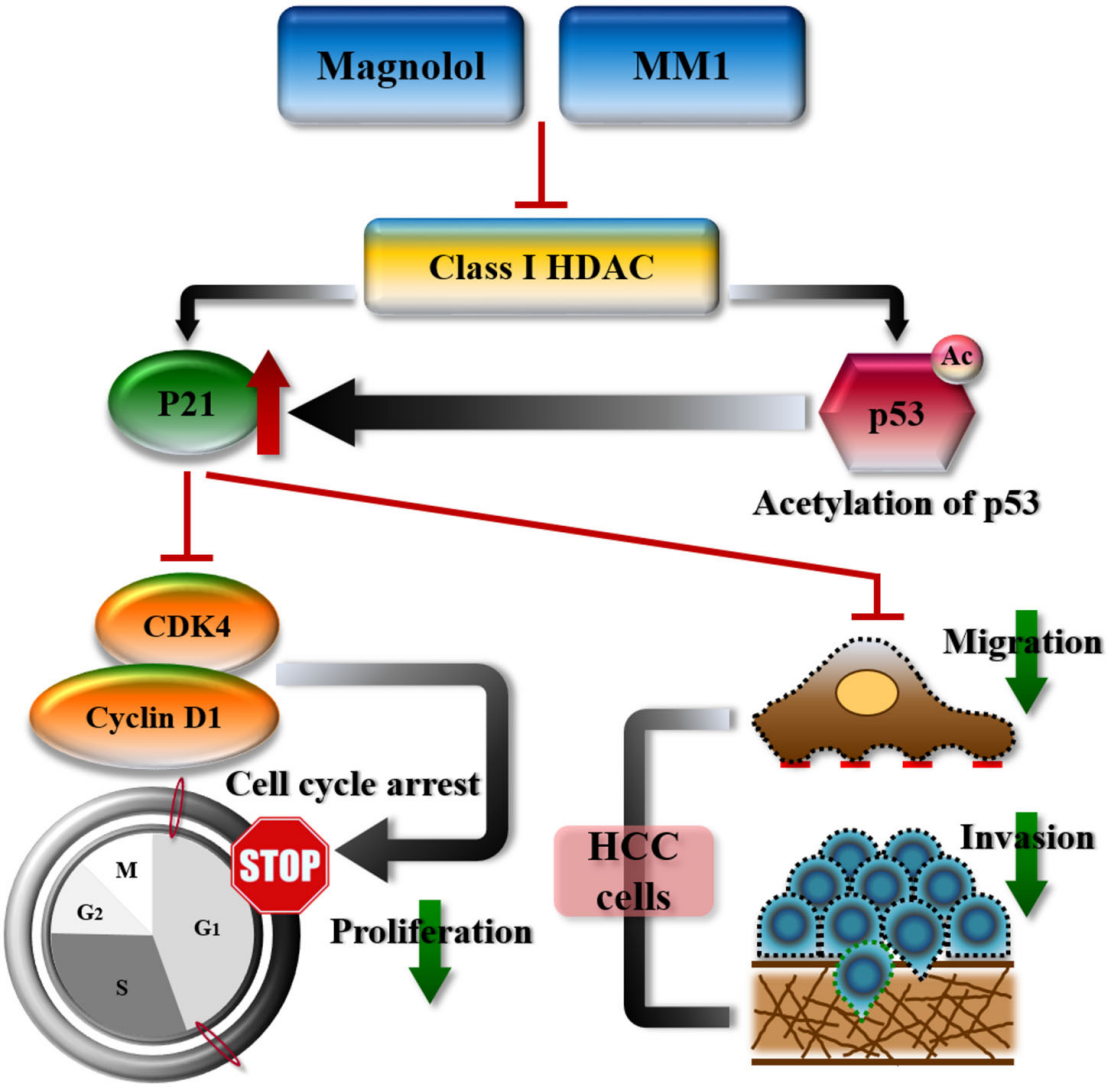
Schematic representation summarizing the anti-hepatocellular carcinoma mechanisms of magnolol or 2-O-methylmagnolol (MM1). MM1 and magnolol inhibited cell cycle progression and tumor growth by inhibiting class I histone deacetylase expression and promoting p21 expression and p53 acetylation.

Mouse tumor tissues were sectioned and subjected to immunohistochemical staining to analyze the expression of class I HDACs and p21, CDK4, cyclin D1, Ki-67, and EMT-related genes. Our results were consistent with those from *in vivo* experiments, that is, dramatic decreases in class I HDACs, CDK4, cyclin D1, Ki-67, and EMT-promoted protein expression and increased p21 and E-cadherin expression in tumor tissues of mice treated with magnolol or MM1 (Figures 6G,H). These findings confirm that magnolol and MM1 induce the expression of the above tumor-suppressor genes by inhibiting class I HDACs, thereby inhibiting HCC growth and metastasis (Figure 7).

## Discussion

In the present study, we tested the anti-HCC effects of magnolol and its methoxylated derivative MM1 and elucidated their modes of action. Both the cell and the animal models showed that magnolol and MM1 inhibited HCC cell and tumor growth, although the inhibitory effect of MM1 was superior to that of magnolol at similar concentrations. Additionally, we found that magnolol and MM1 inhibited cell cycle progression and tumor growth by inhibiting class I HDAC expression and promoting p21 expression and p53 acetylation. To the best of our knowledge, this is the first study to report the anti-HCC activity of MM1 and its superior potential for liver cancer treatment compared to that of magnolol.

Due to their extensive range of gene regulation, HDACs affect multiple physiological processes, including cell growth, differentiation, and apoptosis. Previous studies have suggested that abnormal HDAC expression is closely associated with the occurrence of various diseases, including cancer, and therefore identified HDACs as key therapeutic targets (32, 50, 51). Among HDAC types, substantial expression of class I HDACs is commonly observed in most cancer types, including HDACs 1 and 2 in breast cancer (52, 53); HDAC 1 in lung cancer (54); HDACs 2 and 3 in colorectal cancer (55); and HDACs 1, 2, and 3 in liver cancer (56, 57). Considering this, we focused on the inhibitory effects of magnolol and MM1 on class I HDACs to investigate the feasibility of their clinical applications. However, due to the overexpression of other types of HDACs in other cancer types, it is necessary to analyze the inhibitory effects of these compounds on other types of HDACs to determine whether they can be used for the treatment of other cancers. Furthermore, although we discussed the effects of magnolol and MM1 on the tumor-suppressor genes p21 and p53, these results may only partially explain the anticancer mechanism of magnolol and MM1. Future studies will continue to investigate the effects of these two compounds on the regulation of other tumor-suppressor pathways to better understand the mechanisms by which they act to suppress tumors.

We observed that magnolol and MM1 enhanced p21 expression by inducing histone acetylation, thereby inhibiting cyclin D1 and CDK4 activities, as well as cell cycle progression. Additionally, magnolol and MM1 also induce p53 protein acetylation, which not only enhances its stability but also improves its ability to bind to the target gene promoter, thereby upregulating the expression of downstream tumor-suppressor genes such as p21 and BAX (58). These results indicated that magnolol and MM1 could regulate p21 expression via both direct and indirect pathways and consequently inhibit tumor growth.

Our previous studies confirmed that replacing the hydroxyl functional group of magnolol with a methoxy group could increase the lipophilicity of the methoxylated derivative and improve its skin delivery ability and anti-inflammatory activity (43). In another study, we also confirmed that the same concentration of MM1 could induce increased expression of the tumor suppressor long non-coding RNA, GAS5, compared to magnolol, and exert a greater inhibitory effect on skin cancer cells (59). Consistent with previous research, we found that the same concentration of MM1 could yield better anti-liver cancer activity compared to that of magnolol. This may be due to the better lipophilicity and cell uptake efficiency of MM1 compared to those of magnolol, as it has higher efficacy at the same concentration. This methoxylation could also increase the mucosal absorption rate of the compound, enhancing the flexibility of the route of administration and its clinical applicability.

Multiple clinical trials have shown excellent outcomes for HDAC inhibitors, including chidamide, panobinostat, vorinostat, and SAHA, in the treatment of many cancers (60, 61). Among these, SAHA was the first HDAC inhibitor approved by the FDA for the treatment of T-cell lymphoma. It can specifically bind to the zinc-containing catalytic domains of class I, II, and VI HDACs, thus inhibiting their enzymatic activities. In addition to T-cell lymphoma, SAHA has shown promise in treating cancers of the breast, lungs, and prostate. Additionally, the combined use of HDAC inhibitors with other clinical anticancer medications shows compounded effects (62–64). For example, the combined use of SAHA and bortezomib promotes nasopharyngeal cancer cell apoptosis (65), and the combined use of romidepsin with cisplatin and gemcitabine enhances their therapeutic effects against triple-negative breast cancer (66). In the present study, we found that magnolol and MM1 inhibit the growth of HCC cells by suppressing the expression of class I HDAC, which is different from the mechanism of action of SAHA. However, we also observed that the combined use of magnolol/MM1 and sorafenib substantially enhanced their antiproliferative effects on HCC cells. The findings indicate the potential of using magnolol/MM1 as an adjuvant in combination with sorafenib in liver cancer treatment. Future studies will continue to investigate the optimal combination and dosage of magnolol/MM1 and existing clinical drugs including sorafenib and SAHA.

Sorafenib is an FDA-approved kinase inhibitor that inhibits the activation of tyrosine kinases such as VEGFR, PDGFR, and RAF family kinases (67). It has also been reported to induce the expression of p21 and p53 (68, 69), which is the main tumor suppressor regulatory pathway of magnolol and MM1. Before fully elucidating the interaction between these drugs and molecules, we cannot assume that the additive anti-HCC effect of magnolol/MM1 and sorafenib is entirely due to the activation of the p21 and p53 tumor suppression pathways. However, we believe that these molecules should play an important regulatory role. In addition, we observed that the combined use of magnolol/MM1 and sorafenib not only substantially enhanced their antiproliferative effects on HCC cells but also reduced the toxicity of sorafenib monotherapy in normal cells. Further studies are also required to determine the mechanisms by which magnolol/MM1 reduces the physiological toxicity of sorafenib.

In conclusion, although HDAC inhibitors have been used extensively for the treatment of various cancers, their side effects remain a bottleneck to their clinical application. In this study, we synthesized a methoxylated derivative of magnolol, MM1, and verified its superior anti-HCC activity over magnolol. Additionally, it can enhance the therapeutic effect of sorafenib, when used in conjunction, and does not present physiological toxicity. Thus, MM1 is a suitable combination therapeutic adjuvant to improve the therapeutic efficacy of anticancer drugs.

## Data Availability Statement

The raw data supporting the conclusions of this article will be made available by the authors, without undue reservation.

## Ethics Statement

This animal study was reviewed and approved by the Animal Care Ethics Commission of Chang Gung Memorial Hospital (IACUC Approval No. 2018031301, Approval Date: 6/19/2018).

## Author Contributions

T-HW, J-YF, and C-YC: conceptualization. C-YC and T-HW: data curation. C-CC, W-YC, and Y-LL: formal analysis. T-HW, L-SW, and S-FC: investigation. CH and J-YF: methodology. T-HW, C-CC, and C-YC: project administration. Y-LL and J-YF: resources. T-HW: supervision. S-HU: validation. S-HU, W-YC, and CH: visualization. T-HW and C-YC: writing of the original manuscript draft and manuscript review and editing. All authors contributed to manuscript revision and have read and approved the submitted version.

## Conflict of Interest

The authors declare that the research was conducted in the absence of any commercial or financial relationships that could be construed as a potential conflict of interest.
